# Does Work Disability Contribute to Trajectories of Work Participation before and after Vocational Labour Market Training for Job Seekers?

**DOI:** 10.3390/ijerph18031347

**Published:** 2021-02-02

**Authors:** Taina Leinonen, Eira Viikari-Juntura, Heikki Räisänen, Santtu Sundvall, Antti Kauhanen, Svetlana Solovieva

**Affiliations:** 1 Finnish Institute of Occupational Health, 00032 Helsinki, Finland; eira.viikari-juntura@ttl.fi (E.V.-J.); svetlana.solovieva@ttl.fi (S.S.); 2 Ministry of Economic Affairs and Employment, 00023 Helsinki, Finland; heikki.raisanen@tem.fi (H.R.); santtu.sundvall@tem.fi (S.S.); 3 Etla Economic Research, 00100 Helsinki, Finland; antti.kauhanen@etla.fi

**Keywords:** active labour market programme, disability retirement, latent groups, occupation, open labour market, paid employment, public employment services, register study, sickness absence, unemployment

## Abstract

The contribution of ill-health to labour market participation in relation to vocational training is unclear. Using nationally representative Finnish register data on 42,691 vocational labour market trainees in 2008–2010, we constructed latent trajectory groups of work participation in the open labour market three years before and after training, identifying groups called “High–High”, “High–Low”, “Low–High”, and “Low–Low”. We plotted further patterns of labour market participation within these trajectory groups and, using multinomial logistic regression, examined assignment to these groups focusing on previous work disability status. Those with compared to those without previous work disability had previous employment more often and spent less time in economic inactivity within the two trajectory groups with low pre-training levels of work participation. Having a previous work disability was associated with assignment to the “High–Low” trajectory group of work participation instead of the “High–High” comparison group. The associations of other background factors with the assignment to the different trajectory groups were relatively similar amongst those with and without previous work disability. However, some of these associations were weaker amongst the former. Along with other key background factors, previous work disability should be accounted for when assessing the effects of vocational training.

## 1. Introduction

People with health problems have lower employment rates [1,2,3], a higher likelihood of employment exit through unemployment or other routes [4,5,6,7,8,9], and a lower likelihood of re-employment [4,10,11,12,13,14,15,16] than people without these problems. Health issues have, therefore, received special attention with respect to interventions promoting work participation [1,17,18,19,20]. Previous studies have indicated that health also influences outcomes of interventions that are not primarily health-related, such as in active labour market programmes [21,22,23].

Along with some other active labour market programmes, training has a good potential to increase employment participation [24,25,26,27]. Individuals may enter training with large differences in health status, sociodemographic characteristics, and labour market history, which may influence subsequent labour market outcomes and the effectiveness of the programmes [25,26]. Relatively little is known about the contribution of health to labour market outcomes in relation to the training provided as an active labour market programme. Previous Danish findings indicated that ordinary education and subsidised job training had positive effects on employment in an originally sick-listed employed population [28]. It nevertheless remained unclear how the group would have fared in comparison to programme participants without sick-listing.

Individuals with previous work disability related to, e.g., long-term sickness absence or disability pension are likely to have specific circumstances that influence training outcomes. On the one hand, people with previous work disability have experienced reduced work ability due to medically certified health conditions, which may limit the success of training in promoting work participation. On the other hand, having been absent from work may reflect a relatively good attachment to the labour market, which may facilitate work resumption after training. As a result, individuals with previous work disability may have simultaneous disadvantages and advantages compared to other training participants.

The contribution of health problems and work disability to labour market outcomes in relation to the training provided as an active labour market programme remains unclear. Information on the influence of health-related and other background factors on labour market participation over periods of several years before and after training would provide important insights into the factors that may play an important role in the effectiveness of training programmes. In the present study, we examined whether previous work disability contributes to long-term trajectories of work participation in the open labour market before and after vocational labour market training in Finland. We aimed to answer the following research questions.

1. What are typical trajectories of work participation over a period of several years before and after vocational labour market training?
2. Within the identified trajectory groups of work participation, what are the further patterns of labour market participation (such as unemployment, participation in active labour market programmes, and economic inactivity) amongst those with and without a previous work disability?
3. How is previous work disability status associated with following the identified trajectory groups of work participation?
4. Does the association of other background factors with following the trajectory groups of work participation vary by previous work disability status?

## 2. Materials and Methods

### 2.1. Data

We used a 70% random sample of the working-age population living in Finland on the last day of 2007. Individual-level register-based data were available both retrospectively and prospectively. Information was obtained on episodes of vocational labour market training and other active labour market programmes, episodes of unemployment as well as sociodemographic and employment-related factors from the Finnish Longitudinal Employer‒Employee Data (FLEED), on episodes of employment, earnings-related retirement, and other benefit receipt from the Finnish Centre for Pensions, and episodes of compensated sickness absence and national pensions from the Finnish Social Insurance Institution.

In Finland, vocational labour market training (vocational training in short) is provided as a part of nationwide public employment services. The scheme is different from training that may be included as a part of vocational rehabilitation within the earnings-related pension scheme, which has been studied elsewhere [29,30]. Whilst vocational rehabilitation is provided to individuals who are relatively well attached to the labour market who have a threat of work disability due to an illness or injury, vocational training is provided to job seekers on a non-health basis. Vocational training can take on very different forms ranging between short courses and completion of vocational degrees or their parts.

In this study, we included individuals who had incident vocational training at age 25–54 in 2008, 2009, or 2010 (originally 49,072 individuals). Incident vocational training was determined as the first episode occurring in one of these years without any vocational training in the preceding two calendar years. A study person could nevertheless have participated in other active labour market programmes, including, e.g., preparatory labour market training, subsidised employment, work trials, or practical training. When determining the dates of onset and termination of vocational training, successive episodes with time gaps of ≤32 days were combined. For determining the duration of training, however, these time gaps were not included in the calculation. Duration of training was categorised as ≤0.4 months (1–12 days), >0.4–2 months, >2–6 months, >6–12 months, and >12 months.

We excluded first-generation immigrants (*n* = 4492, 9.2%) because this group may participate in specific types of labour market programmes. We also excluded individuals who received permanent full pensions (*n* = 59, 0.1%) because this group was unlikely to return to normal work duties. To apply the full three years’ follow-up periods before the onset and after the termination of vocational training, we further excluded individuals who were not living in Finland in the three preceding calendar years (*n* = 199, 0.4%) or whose training lasted beyond 31 October 2011 (*n* = 1631, 3.3%). Follow-up information was available until 31 October 2014. The final study population consisted of 42,691 individuals.

### 2.2. Trajectories of Work Participation

Information on labour market participation was based on episodes of employment, unemployment, participation in active labour market programmes, and receipt of other benefits, available on a day-to-day basis. Work participation was assessed as work in the open labour market, i.e., being in paid employment without participating in active labour market programmes and without receiving unemployment or work disability benefits. We calculated the proportion of time per month that was spent in work over the period covering three years before and three years after vocational training. Work participation trajectories were examined to capture temporal patterns in the level of participation in competitive work over time in relation to vocational training, taking into account that exiting and re-entering work may constitute dynamic processes instead of single events. Work participation trajectories have also been previously examined in studies focusing on outcomes of work-related interventions [21,22,29,31].

For the main outcome of this study, we constructed latent trajectories of work participation, assessing the analysis time as a single six-year period, excluding the time spent in training. The trajectories were obtained using a semiparametric group-based modelling strategy with the normal distribution as the underlying statistical model [32,33]. The Bayesian information criterion (BIC) was considered when selecting the optimal model, number of trajectories and their shape. The analyses were carried out using the PROC TRAJ module for SAS version 9.4 (SAS Institute, Cary, NC, USA).

### 2.3. Further Labour Market Participation

Within the constructed trajectory groups of work participation, we examined further labour market participation before and after vocational training by plotting changes in the proportion of time per month that was spent in six mutually exclusive statuses: (1) work in the open labour market (basis for the trajectory groups of work participation as described above), (2) employment with active labour market programmes (participating in active labour market programmes whilst being in paid employment, including subsidised employment), (3) unemployment with active labour market programmes (participating in active labour market programmes without being in paid employment), (4) unemployment with active job seeking (being an unemployed or laid-off job seeker without participating in active labour market programmes) (5) time-restricted work disability (receiving full or part-time compensated sickness allowance, temporary or partial disability pension, or vocational rehabilitation), and (6) economic inactivity (other statuses outside the labour force).

We plotted the changes in the proportion of time spent in different labour market statuses separately for those with and without previous work disability. For previous work disability status, we determined whether a study person had time-restricted work disability, i.e., was in the abovementioned status 5 of labour market participation, for at least one day during the three-year period preceding vocational training. The measure was thereby based on having received temporary or partial work disability benefits or services. During sick leave, permanent Finnish residents can receive sickness allowance compensated by the Social Insurance Institution of Finland after a waiting period of 10 working days that are typically paid by the employer. Full sickness allowance is paid for a maximum of 300 working days. A part-time sickness allowance is a voluntary option for employees who have been assessed by a physician as incapable of performing their regular work duties but have been able to return to part-time work performing 40–60% of regular hours. A temporary disability pension may be granted in the case of longer-term loss of work ability by at least 60% that is nevertheless still expected to be restored. Temporary or permanent partial disability pension may be granted if work ability has reduced by 40–60%. Vocational rehabilitation of the earnings-related pension scheme may be granted to individuals who have a recent attachment to the labour market, who have a threat of disability retirement within the next five years due to an illness or injury, and whose work participation can be expected to be promoted and disability retirement postponed or prevented with rehabilitation.

### 2.4. Background Factors

We examined various background factors as potentially associated with assignment to the constructed trajectory groups of work participation. Previous work disability was included as a dichotomous variable as described above.

We pooled men and women in the main analyses. Compared to many other countries, the gender difference in employment participation in Finland is relatively small, with women having high participation rates, particularly in full-time employment. At the same time, however, occupational and sectoral gender segregation in the labour market is relatively strong [34]. We, therefore, present supplementary descriptive information of the study population by gender. Furthermore, we examined the interaction between previous work disability and other background factors, including gender.

Age was categorised at five-year intervals. Region of residence (Southern, Western, Eastern, and Northern Finland) and education (tertiary, secondary, and primary) were measured at the end of the calendar year preceding vocational training. We examined years passed since the highest completed educational level in categories 1–2, 3–5, 6–10, 11–20, and >20. For secondary and tertiary education, this information was derived based on the year of the completed degree. For primary education, this information was approximated based on age, as primary education is typically completed in the year of one’s 16th birthday.

Factors related to occupational history were determined for those who were employed during the last week of at least one of the calendar years before vocational training for which the occupational information was available. The most recent information was used, available for occupational class (upper non-manual, lower non-manual, skilled manual, unskilled manual, and self-employed) since 2004 and the employment sector (private and public) and industrial sector since 2003. The industrial sector included the categories: (1) manufacturing, (2) construction, (3) trade (wholesale and retail trade; repair of motor vehicles and motorcycles), (4) transportation and storage, (5) knowledge work (information and communication; financial and insurance activities; real estate activities; professional, scientific and technical activities), (6) human health and social work activities, and (7) other. The subpopulation consisting of those with occupational history, i.e., those for whom information on each of the three occupational factors was determined, included 37,638 individuals (88.2%).

### 2.5. Regression Analyses

For examining how previous work disability and other background factors were associated with assignment to the different trajectory groups of work participation before and after vocational training, we used multinomial logistic regression analysis. We calculated relative risk ratios (RRR) of assignment to different trajectory groups and their 95% confidence intervals. We examined the effect of previous work disability amongst the total study population as well as amongst the subpopulation with occupational history. We also tested whether there were statistically significant interactions between previous work disability status and other background factors and present the results for other background factors separately for those with and without previous work disability.

## 3. Results

Amongst the study population consisting of individuals participating in vocational training, 29.7% had previous work disability (Table 1). Those with compared to those without previous work disability were more often older, female and had lower education and more time since completed education. Those with previous work disability more often had previous employment for determining factors related to occupational history, were manual workers, and employed primarily in the manufacturing, or health and social work sectors also. These differences in the distributions by previous work disability status were found amongst both genders, although the proportion of those employed in the health and social work sector was altogether small amongst men (Supplementary Table S1).

**Figure 1:**
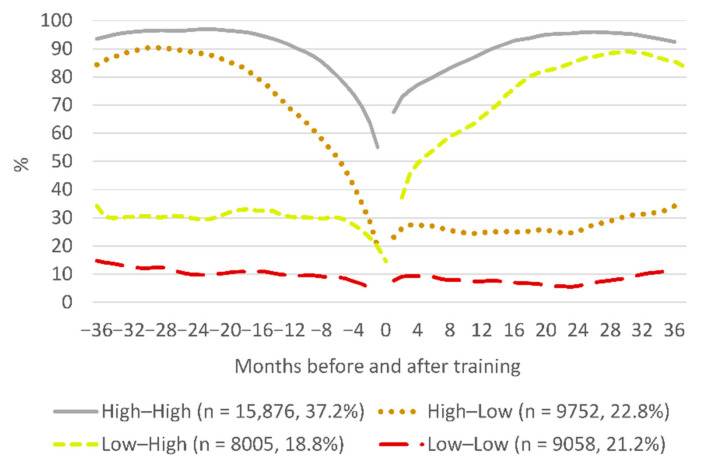
Trajectory groups of work participation in the open labour market before and after vocational labour market training.

Four trajectory groups of work participation were identified (Figure 1). The largest group (37.2%), called the High–High group, participated in work more than 90% of the time until about one year before vocational training, after which work participation reduced, falling to less than 60% by the time the training started. After training, the level of work participation increased, resuming an above 90% level early during the second year after training. The group called High–Low (22.8%) also had a relatively high initial level of work participation, which nevertheless declined to low levels of around 20–30% before training, at which it also remained after training. A similar low level of work participation was initially observed also for the group called Low–High (18.8%), but for this group, work participation increased after training, reaching an almost 90% level during the third year. The group called Low–Low (21.2%) had work participation constantly at a very low level at above or below 10%.

**Figure 2:**
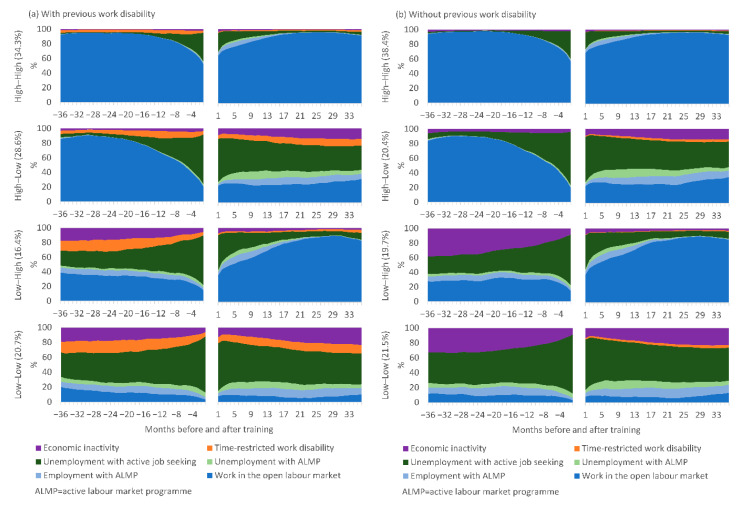
Monthly percentage of time spent in different labour market statuses before and after vocational labour market training amongst the different trajectory groups of work participation in the open labour market, separately for those (**a**) with (*n* = 12,697) and (**b**) without (*n* = 29,994) previous work disability.

Patterns of further labour market participation before and after vocational training are presented for the four different trajectory groups of work participation, separately for those with (Figure 2a) and without (Figure 2b) previous work disability. Those with compared to those without previous work disability had a higher proportion assigned to the High–Low group (28.6% and 20.4%) and lower proportions assigned to the High–High (34.3% and 38.4%) and Low–High (16.4% and 19.7%) groups. Within the trajectory groups, however, patterns of work in the open labour market (the basis for the trajectory groups), employment and unemployment with active labour market programmes, and unemployment with active job seeking were relatively similar amongst those with and without previous work disability. In the two trajectory groups with low pre-training levels of work participation, those with previous work disability spent around 10% of the time in the work disability status before training, whereas those without previous work disability spent a corresponding excess amount of time in economic inactivity.

In the total study population, after adjusting for all other background factors than the factors related to occupational history in Model 1, those with previous work disability had an increased risk of assignment to the High–Low trajectory group of work participation and a somewhat reduced risk of assignment to the Low-High group instead of assignment to the High–High comparison group (Table 2). In the subpopulation with occupational history, those with previous work disability still had a similar increased risk of assignment to the High–Low trajectory group, but no reduced risk of assignment to the Low-High group and a somewhat increased risk of assignment to the Low–Low group. Results for this subpopulation were similar in Model 1 and Model 2, with further adjustment for the factors related to occupational history in the latter. Although the inclusion of these factors improved the predictive value of the model (pseudo R2 increased from 0.049 in Model 1 to 0.084 in Model 2), it remained relatively small. The difference in the results between the total study population and the subpopulation with occupational history can be attributable to the fact that having previous employment for determining occupational history was more common amongst those with (91.9%) than those without (86.6%) previous work disability; in the subpopulation analyses, excessive exclusion of those without previous work disability due to previous non-employment led to an increased representation of those with previous work disability in the Low–High (from 26.0% to 28.4%) and Low–Low (from 29.0% to 32.8%) trajectory groups of work participation.

The results regarding other background factors are presented for those with (Table 3a) and without (Table 3b) previous work disability only in the subpopulation with occupational history to be able to include and mutually adjust for all factors simultaneously. Those who were younger had an increased risk of assignment to the Low–High trajectory group of work participation instead of assignment to the High–High comparison group regardless of previous work disability status, whereas those who were older had an increased risk of assignment to the High–Low group only amongst those without previous work disability (interaction between previous work disability and age was on the verge of statistical significance: *p* = 0.055). Amongst those without previous work disability, women, those employed in the public sector, and generally also those living in other regions than Southern Finland, particularly the North, had an increased risk of assignment to the other three trajectory groups of work participation than the High–High group. Amongst those with previous work disability, the corresponding effects of gender (interaction with previous work disability: *p* = 0.001), employment sector (interaction with previous work disability: *p* = 0.000), and region of residence (interaction with previous work disability: *p* = 0.027) were weaker or even lacking. As a result, the predictive value of the model was smaller amongst those with compared to those without previous work disability (pseudo R2 was 0.068 vs. 0.090, respectively).

No statistically significant interactions were observed between previous work disability and the remaining examined factors (*p* > 0.05). Regardless of previous work disability status, the risk of assignment to the other three trajectory groups of work participation than the High–High comparison group was increased for those with lower education and occupational class, those employed in most other sectors than manufacturing, and those who started training in 2008 instead of 2009 or 2010. Those who had recently completed their education had an increased risk of assignment to the Low–High group. A shorter duration of training was associated with assignment to the two trajectory groups with low post-training levels of work participation.

## 4. Discussion

In nationally representative Finnish register data, much diversity was observed in long-term trajectories of work participation in the open labour market before and after vocational labour market training, provided as a part of public employment services for job seekers. We found that over one-third of the participants in vocational training followed a trajectory where work participation temporarily declined from an initial high level before training and resumed to this high level after training. The other three trajectory groups were of relatively equal size, consisting of one where work participation declined from an initial high level to a low level despite training, one where work participation increased from an initial low level to a high level after training and one where work participation was constantly at a very low level.

We further found that within the trajectory groups of work participation, patterns related to unemployment and participation in other active labour market programmes before and after vocational training were relatively similar amongst those with and without previous work disability. In the two trajectory groups with low pre-training levels of work participation, those without previous work disability spent excess time in economic inactivity before training, corresponding with the time spent in time-restricted work disability amongst those with previous work disability. Related to this, vocational trainees with previous work disability more often had occupational histories; time-restricted work disability often relates to having at least some recent employment and can also co-occur with employment periods, e.g., during sickness absence.

Amongst those with previous work disability, relatively good attachment to the labour market may have mitigated some of the negative effects that their health problems had on work participation. Our findings nevertheless indicated that the group appeared to experience also disadvantage in terms of having a high likelihood of following the trajectory where initial high-level work participation declined to a low level after vocational training. Declining health status was likely to be the primary factor driving this trajectory amongst those with previous work disability, whereas amongst those without previous work disability, older age and other potential factors played a more important role. Ill-health and work disability were found to contribute to employment trajectories also in the context of another active labour market programme in Finland, i.e., a subsidised employment programme amongst the long-term unemployed; chronic diseases and sickness absence during the programme increased the likelihood of belonging in poorer trajectories showing weak or declining employment over the follow-up years after the programme [21,22].

Individuals with previous work disability constituted a surprisingly large group amongst our study population of vocational labour market trainees, their proportion being around 30%. The question remains whether the provided training was an appropriate intervention for these individuals or whether they would have required other types of services addressing the potentially persisting problems of work ability. Furthermore, it remains unclear to what extent health limitations were identified and addressed by the employment services and whether this was done in a timely manner. Problems of health and work ability have been recognised as key factors amongst the multiple and often co-occurring employment barriers in Finland [35]. Considerations of ill-health and work disability should be better accounted for also when assessing the effectiveness of vocational training and other active labour market programmes.

We found that regardless of previous work disability status, those who were younger or had recently completed their education commonly followed the trajectory where initial low-level work participation increased to a high level after vocational training. This type of trajectory appears to reflect successful entry into the labour market. Without a control group, however, we cannot determine whether this favourable outcome was caused by vocational training. Previous study results on vocational labour market training in Finland [36] or active labour market programmes more generally [26] do not suggest that the interventions would be more effective amongst the young.

According to our findings, amongst both those with and without previous work disability, a shorter duration of vocational training was associated with following the two trajectories with low post-training levels of work participation. Less favourable work participation outcomes in shorter training can reflect the smaller effectiveness of shorter programmes, but also, e.g., selection of individuals with poorer work prospects into participating in shorter programmes or dropping out from training.

We also found that the likelihood of following each of the other three trajectories than the most favourable, i.e., where initial high-level work participation was resumed after vocational training, was generally increased for women, those living in other regions than Southern Finland, those with lower education and occupational class, those employed in the public sector and most industrial sectors other than manufacturing, and those who started vocational training before the onset year of the most recent economic recession in 2009. However, the associations of gender, region of residence, and employment sector were weaker amongst those with compared to those without previous work disability. It may be that amongst those with previous work disability, problems of health and work ability largely affect work participation trajectories resulting in a generally smaller contribution of some other background factors. The weaker contribution of gender, region of residence, and employment sector amongst those with previous work disability may also be attributable to specific reasons, discussed below.

In our data, previous work disability was more common amongst women than amongst men. Furthermore, previous studies have indicated that men have fewer contacts with health care services than women [37,38,39]. Men may, therefore, have poorer or delayed access to treatment and be less prone to apply for work disability benefits. Consequently, men who do receive these benefits may be excessively selected in terms of ill-health. This may explain why in our study, men’s advantage over women with respect to work participation was less pronounced amongst those with previous work disability than amongst vocational trainees more generally.

In Finland, the Southern region includes the more populated capital area that has higher employment rates than other regions [40]. This is likely to explain the association of living in the other regions with less favourable work participation trajectories. Moreover, the circumstance of having experienced work-related disability may reflect labour market attachment to a larger extent in areas with poorer than in areas with better overall employment opportunities, which may potentially explain the attenuated regional differences in work participation trajectories amongst the vocational trainees with previous work disability.

Employment careers have typically been perceived as more secure in the public than in the private sector. However, in our study population consisting of job seekers, employment history in the public sector was actually associated with following less favourable work participation trajectories. The effect of employment sector being weaker amongst those with previous work disability may relate to differences between the employment sectors in the definition of work disability. In the private sector, the definition is based on general opportunities to perform work that can be reasonably expected, taking into account the person’s background. In the public sector, work disability is defined on an occupational basis considering performance in the person’s own work. This could mean that in our data, work disability of those who had employment history in the public sector was more often occupation-specific, leaving opportunities for being able to perform other types of work tasks, whereas work disability of those coming from the private sector was more comprehensive. This might explain the attenuated differences between the private and the public sector in work participation trajectories amongst the vocational trainees with previous work disability.

Our finding on the association of lower education and occupational class with following less favourable work participation trajectories around vocational training—irrespective of previous work disability status—is in accordance with socioeconomic differences in labour market outcomes that are typically found in general populations [41,42]. It is less clear why following less favourable work participation trajectories was found to be more common in most other sectors than manufacturing. This sector is known to have been hit particularly hard by the economic recession peaking in 2009 [40]. As a result, job seekers coming from the manufacturing sector compared to those coming from other sectors may have been more likely to experience employment problems due to abrupt macroeconomic hardship rather than a long-term individual disadvantage. This would also be in line with our finding indicating that the work participation trajectories were less favourable amongst those starting vocational training before the recession; during normal economic conditions, selection into unemployment is more likely to be driven by disadvantageous individual characteristics than in times of economic recession during which job loss is more widespread [43,44].

Our research data and methods had several strengths. The nationally representative study population comprised job seekers participating in vocational labour market training provided as a part of public employment services. The register-based data did not have the problem of non-response or loss to follow-up. Moreover, in addition to sociodemographic and occupational information, the rich data included episode information on employment, benefit receipt, and participation in active labour market programmes, based on which we could define day-by-day statuses of labour market participation. By using a semiparametric group-based modelling strategy, we provided novel findings on latent trajectory groups of work participation in the open labour market before and after vocational training.

There were nevertheless also some limitations. Even though previous work disability status, as well as various sociodemographic and work-related factors, were included in the analyses, the predictive capacity of the models remained relatively small. One of the shortages of register-based data is that they do not include information on personal characteristics, such as work motivation, lifestyle factors or self-assessed health, which may influence work opportunities and decisions of the individuals. The predictive value of the model was weaker amongst those with compared to those without previous work disability. Amongst those with work disability, health is likely to play a particularly important role in predicting work participation trajectories, but not all health aspects could be captured with the available data. Moreover, we could not fully address the differences in health and work ability between those with and without previous work disability. It is likely that, e.g., those who were outside the labour market had health conditions that never led to work disability. As a result, our findings may not apply to the contribution of all types of health conditions to trajectories of work participation around vocational training.

Our data did not either have information on the content of vocational labour market training. The type of training and the occupational qualifications targeted by training may vary considerably within the provided service. Moreover, the content of vocational training may have changed from the period of our study. After 2010 the overall use of vocational training started to decline, whilst at the same time its use increased amongst those with recent participation in work or education. There have also been changes in some of the other types of active labour market programmes, which may have influenced the patterns of use of vocational training [45]. Furthermore, structural changes in the labour market may have affected the demand for certain occupational qualifications and, thereby, the content of the provided training. These factors may have influenced the labour market outcomes of vocational trainees. However, our main finding showing the importance of previous work disability on work participation trajectories in relation to vocational training is likely to apply more widely than to a single national system at a particular point in time.

It should also be kept in mind that our findings on the variation in work participation trajectories before and after vocational training do not provide information on the effectiveness of training. Groups with beneficial background characteristics are likely to have good employment opportunities irrespective of whether they participate in further training. The groups that have the most favourable work participation trajectories are, therefore, not necessarily the ones that benefit most from the intervention.

## 5. Conclusions

Work disability contributed to trajectories of work participation in the open labour market and further labour market participation before and after vocational labour market training. Although those with previous work disability had an advantage in terms of more often having employment history, they also experienced a disadvantage in terms of more often following a trajectory where initial high-level work participation declined to a low level after vocational training. Overall, background factors that were associated with following the different work participation trajectories were relatively similar amongst those with and without previous work disability. However, some sociodemographic factors, such as female gender and public sector employment, were more strongly associated with following the less favourable work participation trajectories amongst those without previous work disability.

Future studies should assess whether the effectiveness of vocational labour market training on work participation varies depending on previous work disability in combination with other key background factors, including personal circumstances of the individuals. This information would help determine whether the vocational training provided within regular employment services is an appropriate intervention for different population groups with problems in health and work ability. If not, other types of interventions may be required, e.g., more individually tailored services that would ensure that the target occupation is less demanding in terms of the physical and psychosocial work exposures that likely had previously contributed to reduced work ability.

## Figures and Tables

**Table 1:** Distribution of background factors amongst the study population of vocational labour market trainees with and without previous work disability.

Background Factors	With Previous Work Disability		Without Previous Work Disability		*p*-Value for Difference in the Distribution
	*n*	%	*n*	%	
Age					0.000
25–29	1800	14.2	6566	21.9	
30–34	1917	15.1	5437	18.1	
35–39	1973	15.5	4582	15.3	
40–44	2424	19.1	4974	16.6	
45–49	2392	18.8	4610	15.4	
50–54	2191	17.3	3825	12.8	
Gender					0.000
Men	6797	53.5	17,502	58.4	
Women	5900	46.5	12,492	41.7	
Region of residence					0.000
South	5743	45.2	13,391	44.7	
West	3248	25.6	7028	23.4	
East	2053	16.2	4902	16.3	
North	1653	13.0	4673	15.6	
Education					0.000
Tertiary	2543	20.0	8441	28.1	
Secondary	7377	58.1	16,039	53.5	
Primary	2777	21.9	5514	18.4	
Years since completed education					0.000
1–2	515	4.1	2096	7.0	
3–5	999	7.9	3032	10.1	
6–10	2126	16.7	6090	20.3	
11–20	3804	30.0	9127	30.4	
>20	5253	41.4	9649	32.2	
Occupational class					0.000
Upper non-manual	1028	8.1	3654	12.2	
Lower non-manual	3223	25.4	7755	25.9	
Skilled manual	5584	44.0	11,567	38.6	
Unskilled manual	1593	12.6	2809	9.4	
Self-employed	393	3.1	1050	3.5	
No determined occupation	876	6.9	3159	10.5	
Employment sector					0.000
Private	9999	78.8	23,081	77.0	
Public	1897	14.9	3693	12.3	
No determined employment sector	801	6.3	3220	10.7	
Industrial sector					0.000
Manufacturing	4001	31.5	8430	28.1	
Construction	905	7.1	2223	7.4	
Trade	1167	9.2	2865	9.6	
Transportation and storage	726	5.7	1335	4.5	
Knowledge work	1015	8.0	3182	10.6	
Health and social work	1309	10.3	2160	7.2	
Other	2773	21.8	6579	21.9	
No determined industrial sector	801	6.3	3220	10.7	
Start year of training					0.000
2008	4164	32.8	8871	29.6	
2009	4712	37.1	11,135	37.1	
2010	3821	30.1	9988	33.3	
Duration of training in months					0.008
≤0.4	1788	14.1	4204	14.0	
>0.4–2	2270	17.9	5389	18.0	
>2–6	3206	25.3	7700	25.7	
	>6–12	3656	28.8	8883	29.6
>12	1777	14.0	3818	12.7	
Total	12,697	100.0	29,994	100.0	

**Table 2:** Percentage distributions within the trajectory groups of work participation in the open labour market before and after vocational labour market training and relative risk ratios (RRR) and 95% confidence intervals (CI) for being assigned to the trajectory groups by previous work disability status.

Previous Work Disability Status	High–High	High–Low			Low–High			Low–Low		
		(vs. High–High)			(vs. High–High)			(vs. High–High)		
	%	%	RRR	95% CI	%	RRR	95% CI	%	RRR	95% CI
**Total study population**										
Model 1										
Without previous work disability (*n* = 29,994)	72.5	62.8	1.00		74.0	1.00		71.0	1.00	
With previous work disability (*n* = 12,697)	27.5	37.2	1.47	(1.39–1.55)	26.0	0.92	(0.87–0.98)	29.0	0.97	(0.91–1.03)
**Subpopulation with occupational history**										
Model 1										
Without previous work disability (*n* = 25,969)	72.5	62.5	1.00		71.6	1.00		67.2	1.00	
With previous work disability (*n* = 11,669)	27.5	37.5	1.48	(1.40–1.56)	28.4	1.01	(0.94–1.08)	32.8	1.13	(1.05–1.21)
Model 2										
Without previous work disability			1.00			1.00			1.00	
With previous work disability			1.47	(1.39–1.55)		1.00	(0.94–1.07)		1.14	(1.06–1.22)

Model 1: Adjusted for age, gender, region of residence, education, years since completed education, start year of training, and duration of training; Model 2: Model 1 + adjusted for occupational class, employment sector, and industrial sector; Pseudo R2: 0.061 (Model 1) for the total study population and 0.049 (Model 1) and 0.084 (Model 2) for the subpopulation with occupational history.

**Table 3:** Relative risk ratios (RRR) and 95% confidence intervals (CI) for being assigned to the trajectory groups of work participation in the open labour market before and after vocational labour market training by mutually adjusted background factors amongst those (**a**) with (*n* = 11,669) and (**b**) without (*n* = 25,969) previous work disability in the subpopulation with occupational history.

Background Factors	High–Low		Low–High		Low–Low	
	(vs. High–High)		(vs. High–High)		(vs. High–High)	
	RRR	95% CI	RRR	95% CI	RRR	95% CI
**(a) With previous work disability**						
Age						
25–29	1.00		1.00		1.00	
30–34	1.08	(0.90–1.30)	0.91	(0.74–1.13)	1.08	(0.86–1.35)
35–39	0.97	(0.80–1.17)	0.65	(0.52–0.81)	0.82	(0.64–1.04)
40–44	1.11	(0.91–1.35)	0.66	(0.53–0.84)	1.06	(0.83–1.35)
45–49	1.08	(0.88–1.32)	0.48	(0.37–0.62)	1.07	(0.83–1.38)
50–54	1.13	(0.92–1.40)	0.50	(0.39–0.65)	1.08	(0.83–1.41)
Gender						
Men	1.00		1.00		1.00	
Women	1.11	(1.00–1.23)	1.65	(1.44–1.88)	1.01	(0.89–1.16)
Region of residence						
South	1.00		1.00		1.00	
West	0.98	(0.88–1.09)	1.13	(0.98–1.30)	1.29	(1.11–1.49)
East	0.90	(0.78–1.03)	1.33	(1.13–1.56)	1.39	(1.17–1.64)
North	0.98	(0.85–1.14)	1.30	(1.09–1.56)	1.60	(1.34–1.92)
Education						
Tertiary	1.00		1.00		1.00	
Secondary	1.26	(1.10–1.45)	1.41	(1.20–1.67)	1.85	(1.54–2.22)
Primary	1.84	(1.55–2.18)	2.28	(1.84–2.81)	4.47	(3.59–5.57)
Years since completed education						
1–2	1.00		1.00		1.00	
3–5	1.13	(0.83–1.54)	0.46	(0.33–0.62)	0.81	(0.56–1.17)
6–10	1.17	(0.88–1.56)	0.43	(0.32–0.57)	0.77	(0.55–1.08)
11–20	1.07	(0.81–1.42)	0.44	(0.33–0.58)	0.73	(0.53–1.01)
>20	1.06	(0.79–1.41)	0.46	(0.34–0.62)	0.68	(0.48–0.95)
Occupational class						
Upper non-manual	1.00		1.00		1.00	
Lower non-manual	1.11	(0.92–1.33)	0.96	(0.76–1.20)	1.25	(0.97–1.62)
Skilled manual	1.29	(1.06–1.57)	1.15	(0.90–1.47)	1.73	(1.32–2.27)
Unskilled manual	1.41	(1.13–1.76)	1.21	(0.92–1.58)	2.36	(1.77–3.15)
Self-employed	0.87	(0.63–1.19)	1.01	(0.70–1.45)	1.24	(0.84–1.82)
Employment sector						
Private	1.00		1.00		1.00	
Public	1.25	(1.06–1.47)	1.50	(1.27–1.79)	1.52	(1.28–1.81)
Industrial sector						
Manufacturing	1.00		1.00		1.00	
Construction	1.66	(1.39–1.99)	3.14	(2.48–3.98)	3.87	(3.04–4.93)
Trade	1.22	(1.02–1.45)	2.23	(1.78–2.81)	3.74	(2.95–4.74)
Transportation and storage	1.48	(1.22–1.80)	1.97	(1.50–2.59)	2.54	(1.92–3.34)
Knowledge work	1.26	(1.04–1.51)	2.20	(1.72–2.82)	4.92	(3.84–6.30)
Health and social work	0.68	(0.54–0.85)	3.24	(2.56–4.11)	5.70	(4.44–7.33)
Other	1.32	(1.14–1.52)	3.29	(2.74–3.94)	5.66	(4.68–6.85)
Start year of training						
2008	1.37	(1.22–1.52)	1.39	(1.21–1.60)	1.61	(1.40–1.85)
2009	1.00		1.00		1.00	
2010	1.02	(0.91–1.13)	1.14	(0.99–1.31)	1.03	(0.89–1.19)
Duration of training in months						
≤0.4	1.10	(0.94–1.28)	1.14	(0.93–1.40)	1.25	(1.03–1.50)
>0.4–2	0.88	(0.77–1.01)	0.93	(0.78–1.12)	0.95	(0.80–1.13)
>2–6	1.00		1.00		1.00	
>6–12	0.98	(0.86–1.10)	1.28	(1.09–1.50)	0.79	(0.67–0.92)
>12	0.64	(0.55–0.74)	1.18	(0.98–1.41)	0.38	(0.30–0.47)
**(b) Without previous work disability**						
Age						
25–29	1.00		1.00		1.00	
30–34	0.97	(0.86–1.09)	0.78	(0.70–0.88)	0.95	(0.83–1.09)
35–39	1.09	(0.96–1.24)	0.73	(0.64–0.84)	0.94	(0.81–1.10)
40–44	1.24	(1.08–1.41)	0.64	(0.55–0.74)	1.07	(0.91–1.26)
45–49	1.40	(1.21–1.61)	0.50	(0.43–0.59)	1.06	(0.89–1.27)
50–54	1.64	(1.41–1.90)	0.51	(0.42–0.60)	1.13	(0.94–1.36)
Gender						
Men	1.00		1.00		1.00	
Women	1.15	(1.07–1.24)	2.16	(1.99–2.34)	1.31	(1.20–1.44)
Region of residence						
South	1.00		1.00		1.00	
West	1.01	(0.93–1.09)	1.36	(1.24–1.50)	1.44	(1.30–1.60)
East	0.95	(0.87–1.05)	1.43	(1.28–1.58)	1.56	(1.39–1.75)
North	1.30	(1.18–1.43)	1.85	(1.66–2.06)	1.92	(1.71–2.16)
Education						
Tertiary	1.00		1.00		1.00	
Secondary	1.21	(1.10–1.32)	1.27	(1.15–1.40)	1.96	(1.74–2.21)
Primary	1.93	(1.71–2.18)	2.03	(1.77–2.33)	4.46	(3.84–5.18)
Years since completed education						
1–2	1.00		1.00		1.00	
3–5	1.18	(0.99–1.41)	0.60	(0.51–0.71)	0.88	(0.71–1.10)
6–10	1.14	(0.97–1.34)	0.50	(0.43–0.58)	0.89	(0.73–1.08)
11–20	1.09	(0.92–1.29)	0.56	(0.48–0.65)	0.97	(0.79–1.19)
>20	1.00	(0.83–1.20)	0.52	(0.43–0.62)	0.88	(0.70–1.10)
Occupational class						
Upper non-manual	1.00		1.00		1.00	
Lower non-manual	1.08	(0.96–1.20)	1.05	(0.92–1.19)	1.34	(1.15–1.57)
Skilled manual	1.48	(1.31–1.67)	1.50	(1.31–1.73)	1.95	(1.65–2.31)
Unskilled manual	1.65	(1.41–1.92)	1.78	(1.51–2.10)	2.81	(2.33–3.38)
Self-employed	0.77	(0.63–0.95)	1.25	(1.01–1.55)	1.54	(1.22–1.96)
Employment sector						
Private	1.00		1.00		1.00	
Public	1.37	(1.21–1.55)	2.12	(1.89–2.38)	2.57	(2.27–2.9)
Industrial sector						
Manufacturing	1.00		1.00		1.00	
Construction	1.51	(1.34–1.70)	2.46	(2.11–2.86)	3.46	(2.94–4.06)
Trade	1.22	(1.09–1.38)	2.41	(2.10–2.77)	3.26	(2.77–3.84)
Transportation and storage	1.15	(0.99–1.33)	1.92	(1.61–2.30)	1.79	(1.45–2.21)
Knowledge work	1.34	(1.19–1.50)	2.47	(2.15–2.84)	4.02	(3.42–4.73)
Health and social work	0.95	(0.80–1.13)	3.68	(3.13–4.33)	5.88	(4.90–7.05)
Other	1.51	(1.37–1.67)	3.56	(3.18–3.99)	6.30	(5.53–7.18)
Start year of training						
2008	1.37	(1.26–1.49)	1.53	(1.40–1.68)	1.92	(1.74–2.12)
2009	1.00		1.00		1.00	
2010	1.12	(1.04–1.21)	1.12	(1.03–1.23)	1.21	(1.10–1.33)
Duration of training in months						
≤0.4	1.03	(0.93–1.15)	1.01	(0.89–1.16)	1.22	(1.08–1.39)
>0.4–2	1.00	(0.91–1.10)	0.95	(0.85–1.07)	0.97	(0.86–1.09)
>2–6	1.00		1.00		1.00	
>6–12	0.87	(0.80–0.95)	1.13	(1.02–1.24)	0.71	(0.63–0.79)
>12	0.72	(0.65–0.81)	1.04	(0.92–1.18)	0.46	(0.40–0.53)

Pseudo R2: 0.068 (**a**) and 0.090 (**b**); *p*-values for the interaction between previous work disability and other background factors: 0.055 (age), 0.001 (gender), 0.027 (region of residence), 0.534 (education), 0.727 (years since completed education), 0.367 (occupational class), 0.000 (employment sector), 0.061 (industrial sector), 0.105 (start year of training), 0.094 (duration of training).

## Data Availability

We used register data linked together from Statistics Finland, the Finnish Centre for Pensions, and the Social Insurance Institution of Finland. The authors do not have the permission to share this third-party data. Due to data protection regulations, the data can only be accessed by individual researchers who have obtained permission to use the data through an application process (https://www.findata.fi/en/services/data-permits/).

## Supplementary Materials

The following are available online at https://www.mdpi.com/1660-4601/18/3/1347/s1, Table S1: Distribution (%) of background factors amongst the study population of vocational labour market trainees with and without previous work disability, presented separately for men and for women.

## References

[1] McAllister A., Nylén L., Backhans M., Boye K., Thielen K., Whitehead M., Burström B. (2015). Do ‘flexicurity’ policies work for people with low education and health problems? A comparison of labour market policies and employment rates in Denmark, The Netherlands, Sweden, and the United Kingdom 1990–2010. Int. J. Health Serv..

[2] Geiger B.B., van der Wel K.A., Tøge A.G. (2017). Success and failure in narrowing the disability employment gap: Comparing levels and trends across Europe 2002–2014. BMC Public Health.

[3] Schram J.L.D., Schuring M., Oude Hengel K.M., Burdorf A. (2019). Health-related educational inequalities in paid employment across 26 European countries in 2005–2014: Repeated cross-sectional study. BMJ Open.

[4] Paul K.I., Moser K. (2009). Unemployment impairs mental health: Meta-analyses. J. Vocat. Behav..

[5] Ki M., Kelly Y., Sacker A., Nazroo J. (2013). Poor health, employment transitions and gender: Evidence from the British Household Panel Survey. Int. J. Public Health.

[6] Olesen S.C., Butterworth P., Leach L.S., Kelaher M., Pirkis J. (2013). Mental health affects future employment as job loss affects mental health: Findings from a longitudinal population study. BMC Psychiatry.

[7] Van Rijn R.M., Robroek S.J.W., Brouwer S., Burdorf A. (2014). Influence of poor health on exit from paid employment: A systematic review. Occup. Environ. Med..

[8] Kaspersen S.L., Pape K., Vie G.Å., Ose S.O., Krokstad S., Gunnell D., Bjørngaard J.H. (2016). Health and unemployment: 14 years of follow-up on job loss in the Norwegian HUNT Study. Eur. J. Public Health.

[9] Jetha A., Chen C., Mustard C., Ibrahim S., Bielecky A., Beaton D., Smith P. (2017). Longitudinal examination of temporality in the association between chronic disease diagnosis and changes in work status and hours worked. Occup. Environ. Med..

[10] Schuring M., Burdorf L., Kunst A., Mackenbach J. (2007). The effects of ill health on entering and maintaining paid employment: Evidence in European countries. J. Epidemiol. Community Health.

[11] Schuring M., Robroek S.J.W., Otten F.W.J., Arts C.H., Burdorf A. (2013). The effect of ill health and socioeconomic status on labor force exit and re-employment: A prospective study with ten years follow-up in the Netherlands. Scand. J. Work Environ. Health.

[12] Rueda S., Chambers L., Wilson M., Mustard C., Rourke S.B., Bayoumi A., Raboud J., Lavis J. (2012). Association of returning to work with better health in working-aged adults: A systematic review. Am. J. Public Health.

[13] Skärlund M., Åhs A., Westerling R. (2012). Health-related and social factors predicting non-reemployment amongst newly unemployed. BMC Public Health.

[14] Carlier B.E., Schuring M., van Lenthe F.J., Burdorf A. (2014). Influence of health on job-search behavior and re-employment: The role of job-search cognitions and coping resources. J. Occup. Rehabil..

[15] Nwaru C.A., Nygård C.-H., Virtanen P. (2016). Musculoskeletal pain and re-employment among unemployed job seekers: A three-year follow-up study. BMC Public Health.

[16] Svane-Petersen A.C., Dencker-Larsen S. (2016). The impact of self-reported health and register-based prescription medicine purchases on re-employment chances: A prospective study. SSM Popul. Health.

[17] Vooijs M., Leensen M.C.J., Hoving J.L., Daams J.G., Wind H., Frings-Dresen M.H.W. (2015). Disease-generic factors of work participation of workers with a chronic disease: A systematic review. Int. Arch. Occup. Environ. Health.

[18] Vooijs M., Leensen M.C.J., Hoving J.L., Wind H., Frings-Dresen M.H.W. (2015). Interventions to enhance work participation of workers with a chronic disease: A systematic review of reviews. Occup. Environ. Med..

[19] Cancelliere C., Donovan J., Stochkendahl M.J., Biscardi M., Ammendolia C., Myburgh C., Cassidy J.D. (2016). Factors affecting return to work after injury or illness: Best evidence synthesis of systematic reviews. Chiropr. Man. Ther..

[20] Vlachou A., Stavroussi P., Roka O., Vasilou E., Papadimitriou D., Scaratti C., Kadyrbaeva A., Fheodoroff K., Brecelj V., Svestkova O. (2018). Policy guidelines for effective inclusion and reintegration of people with chronic diseases in the workplace: National and European perspectives. Int. J. Environ. Res. Public Health.

[21] Nwaru C.A., Peutere L., Kivimäki M., Pentti J., Vahtera J., Virtanen P.J. (2017). Chronic diseases as predictors of labour market attachment after participation in subsidised re-employment programme: A 6-year follow-up study. J. Epidemiol. Community Health.

[22] Nwaru C.A., Kivimäki M., Pentti J., Vahtera J., Virtanen P. (2018). Sickness absence in a re-employment program as a predictor of labor market attachment among long-term unemployed individuals: A 6-year cohort study in Finland. Scand. J. Work Environ. Health.

[23] Brown J., Katikireddi S.V., Leyland A.H., McQuaid R.W., Frank J., Macdonald E.B. (2018). Age, health and other factors associated with return to work for those engaging with a welfare-to-work initiative: A cohort study of administrative data from the UK’s Work Programme. BMJ Open.

[24] Brown A.J., Koettl J. (2015). Active labor market programs-employment gain or fiscal drain?. IZA J. Labor Econ..

[25] Crépon B., van den Berg G.J. (2016). Active labor market policies. Annu. Rev. Econom..

[26] Card D., Kluve J., Weber A. (2018). What works? A meta analysis of recent active labor market program evaluations. J. Eur. Econ. Assoc..

[27] Vooren M., Haelermans C., Groot W., Maassen van den Brink H. (2019). The effectiveness of active labor market policies: A meta-analysis. J. Econ. Surv..

[28] Holm A., Høgelund J., Gørtz M., Rasmussen K.S., Houlberg H.S.B. (2017). Employment effects of active labor market programs for sick-listed workers. J. Health Econ..

[29] Leinonen T., Solovieva S., Husgafvel-Pursiainen K., Laaksonen M., Viikari-Juntura E. (2019). Do individual and work-related factors differentiate work participation trajectories before and after vocational rehabilitation?. PLoS ONE.

[30] Leinonen T., Viikari-Juntura E., Husgafvel-Pursiainen K., Juvonen-Posti P., Laaksonen M., Solovieva S. (2019). The effectiveness of vocational rehabilitation on work participation: A propensity score matched analysis using nationwide register data. Scand. J. Work Environ. Health.

[31] Hara K.W., Bjørngaard J.H., Jacobsen H.B., Borchgrevink P.C., Johnsen R., Stiles T.C., Brage S., Woodhouse A. (2018). Biopsychosocial predictors and trajectories of work participation after transdiagnostic occupational rehabilitation of participants with mental and somatic disorders: A cohort study. BMC Public Health.

[32] Nagin D.S. (1999). Analyzing developmental trajectories: A semiparametric, group-based approach. Psychol. Methods.

[33] Jones B.L., Nagin D.S. (2007). Advances in group-based trajectory modeling and an SAS procedure for estimating them. Sociol. Methods Res..

[34] Grönlund A., Halldén K., Magnusson C. (2017). A Scandinavian success story? Women’s labour market outcomes in Denmark, Finland, Norway and Sweden. Acta Sociol..

[35] OECD (2020). Faces of Joblessness in Finland: A People-Centred Perspective on Employment Barriers and Policies.

[36] Alasalmi J., Busk H. (2019). Review of the impact of active labour market policy measures [in Finnish, abstract in English]. Finn. Labour Rev..

[37] Vaidya V., Partha G., Karmakar M. (2012). Gender differences in utilization of preventive care services in the United States. J. Womens Health Larchmt.

[38] Wang Y., Hunt K., Nazareth I., Freemantle N., Petersen I. (2013). Do men consult less than women? An analysis of routinely collected UK general practice data. BMJ Open.

[39] Osika Friberg I., Krantz G., Määttä S., Järbrink K. (2016). Sex differences in health care consumption in Sweden: A register-based cross-sectional study. Scand. J. Public Health.

[40] Statistics Finland StatFin Online Service. http://www.stat.fi/tup/statfin/index_en.html.

[41] Carr E., Fleischmann M., Goldberg M., Kuh D., Murray E.T., Stafford M., Stansfeld S., Vahtera J., Xue B., Zaninotto P. (2018). Occupational and educational inequalities in exit from employment at older ages: Evidence from seven prospective cohorts. Occup. Environ. Health.

[42] Schuring M., Schram J.L.D., Robroek S.J.W., Burdorf A. (2019). The contribution of health to educational inequalities in exit from paid employment in five European regions. Scand. J. Work Environ. Health.

[43] Martikainen P., Mäki N., Jäntti M. (2007). The effects of unemployment on mortality following workplace downsizing and workplace closure: A register-based follow-up study of Finnish men and women during economic boom and recession. Am. J. Epidemiol..

[44] Heggebø K., Dahl E. (2015). Unemployment and health selection in diverging economic conditions: Compositional changes? Evidence from 28 European countries. Int. J. Equity Health.

[45] Aho S., Tuomala J., Hämäläinen K., Mäkiaho A. (2018). Targeting of Jobseeker Services and the Later Employment of Participants [in Finnish, Abstract in English].

