# Supplementary material for: Does Work Disability Contribute to Trajectories of Work Participation before and after Vocational Labour Market Training for Job Seekers?
Source: Int J Environ Res Public Health. 2021 Feb 2;18(3):1347. doi: 10.3390/ijerph18031347 (PMC7908399; doi:10.3390/ijerph18031347)
Supplement: Supplementary file 1 [file ijerph-18-01347-s001.pdf]

Table S1. Distribution (%) of background factors amongst the study population of vocational labour market trainees with and without previous work disability, presented separately for men and for women.

Background factors	Men			Women		
	**With Previous Work Disability**	**Without Previous Work Disability**	***p*-Value for Difference in the Distribution**	**With Previous Work Disability**	**Without Previous Work Disability**	***p*-Value for Difference in the Distribution**
Age			0.000			0.000
25–29	15.8	23.3		12.3	19.9	
30–34	15.7	18.2		14.4	18.1	
35–39	15.5	14.7		15.6	16.0	
40–44	18.5	15.7		19.8	17.8	
45–49	18.5	15.1		19.3	15.8	
50–54	16.1	13.0		18.6	12.4	
Region of residence			0.000			0.001
South	44.2	44.3		46.5	45.2	
West	26.4	23.3		24.6	23.6	
East	16.0	16.1		16.4	16.6	
North	13.5	16.3		12.5	14.6	
Education			0.000			0.000
Tertiary	14.8	24.8		26.0	32.8	
Secondary	62.3	56.4		53.3	49.3	
Primary	22.9	18.8		20.7	17.8	
Years since completed education			0.000			0.000
1–2	3.5	6.4		4.6	7.7	
3–5	6.7	9.5		9.2	10.9	
6–10	16.1	20.1		17.5	20.6	
11–20	30.3	30.4		29.6	30.4	
>20	43.3	33.5		39.2	30.3	
Occupational class			0.000			0.000
Upper non-manual	7.9	13.0		8.4	11.1	
Lower non-manual	13.7	17.1		38.9	38.1	
Skilled manual	59.1	48.3		26.5	24.9	
Unskilled manual	9.2	7.6		16.4	11.8	
Self-employed	2.8	3.1		3.4	4.1	
No determined occupation	7.3	10.9		6.4	10.0	
Employment sector			0.000			0.000
Private	86.3	84.0		70.1	67.1	
Public	7.6	7.0		23.4	19.8	
No determined employment sector	6.1	9.1		6.5	13.1	
Industrial sector			0.000			0.000
Manufacturing	42.0	36.5		19.4	16.4	
Construction	12.0	11.4		1.5	1.8	
Trade	7.5	7.7		11.1	12.2	
Transportation and storage	8.0	5.8		3.1	2.6	
Knowledge work	6.8	10.8		9.4	10.3	
Health and social work	2.4	2.3		19.4	14.1	
Other	15.2	16.5		29.5	29.6	
No determined industrial sector	6.1	9.1		6.5	13.1	
Start year of training			0.000			0.001
2008	29.8	26.5		36.2	33.9	
2009	38.8	38.7		35.2	34.9	
2010	31.4	34.8		28.6	31.2	
Duration of training in months			0.298			0.023
≤0.4	17.5	17.1		10.2	9.7	

>0.4–2	18.7	18.8	16.9	16.8
>2–6	24.9	25.2	25.6	26.4
>6–12	26.0	26.9	32.1	33.4
>12	12.9	12.1	15.2	13.6
Total	100.0	100.0	100.0	100.0
N	6 797	17 502	5 900	12 492

3
